# Intra- and Extra-Hospital Dissemination of IMP-22-Producing *Klebsiella pneumonia*e in Northern Portugal: The Breach of the Hospital Frontier Toward the Community

**DOI:** 10.3389/fmicb.2021.777054

**Published:** 2021-12-14

**Authors:** Daniela Gonçalves, Pedro Cecílio, Alberta Faustino, Carmen Iglesias, Fernando Branca, Alexandra Estrada, Helena Ferreira

**Affiliations:** ^1^Microbiology Laboratory - Biological Sciences Department, Faculty of Pharmacy, University of Porto, Porto, Portugal; ^2^UCIBIO - Research Unit on Applied Molecular Biosciences, REQUIMTE, Porto, Portugal; ^3^ISAVE - Instituto Superior de Saúde, Amares, Portugal; ^4^CICS - Interdisciplinary Centre in Health Sciences, Amares, Portugal; ^5^Clinical Pathology Service - Braga Hospital, Braga, Portugal

**Keywords:** *Klebsiella pneumoniae*, antimicrobial resistance, carbapenemases, metallo-β-lactamases, nosocomial infections, intestinal colonization, long-term care facilities

## Abstract

The emergence of infections (and colonization) with *Enterobacteriaceae-*producing carbapenemases is a threatening public health problem. In the last decades, we watched an isolated case becoming a brutal outbreak, a sporadic description becoming an endemic problem. The present study aims to highlight the dissemination of IMP-22-producing *Klebsiella pneumoniae* in the North of Portugal, through the phenotypic and genotypic characterization of isolates collected from hospitalized patients (*n*=5) and out-patients of the emergency ward of the same acute care hospital (*n*=2), and isolates responsible for the intestinal colonization of residents in a Long-Term Care Facility (*n*=4). Pulsed-field gel electrophoresis (PFGE) results, associated with conjugation experiments pointed to a pattern of both vertical and horizontal dissemination. Overall, and complementing other studies that give relevance to IMP-22-producing *K. pneumoniae* in the clinical settings, here we show for the first time the public health threatening breach of the hospital frontier of this resistance threat, toward the community.

## Introduction

Antimicrobial resistance is among the major public health problems of the 21st century. In 2015 the World Health Organization launched the “Global Action Plan on Antimicrobial Resistance” (giving great relevance to antibiotics) to respond to this global issue on five fronts (WHO, 2015). Still, each year, only in the United States and the European Union, 50,000 individuals die due to antibiotic therapy failure (CDC, 2019). Even considering the “last-line” treatment options for infections caused by resistant *Enterobacteriaceae*, carbapenems, we know today several enzymes that can effectively hydrolyze their β-lactam ring, and consequently, compromise their activity (Queenan and Bush, 2007).

Carbapenemases can be divided into two different groups according to their dependency on cations for enzyme activity: serine/non-metallo- (zinc-independent; classes A, C, and D) and metallo-carbapenemases (MBLs; zinc-dependent; class B; Queenan and Bush, 2007). Within the latter, a versatile family of beta-lactamases often associated with *Enterobacteriaceae*, the VIM, IMP, and NDM types are the most relevant carbapenemases globally (Poirel et al., 2011; Nordmann, 2014). Interestingly VIM and IMP are so well settled, that they are considered an endemic problem in the Mediterranean basin (Poirel et al., 2011; Pitout et al., 2015). However, in Portugal, the occurrence of MBL-producing *Enterobacteriaceae* in the clinical settings is apparently not common; only a few sporadic cases were reported, including a VIM-34-producing *Klebsiella pneumoniae* (Rodrigues et al., 2014) and a VIM-2-producing *Klebsiella oxytoca* (Conceicao et al., 2005). In fact, recent studies confirmed that among carbapenemase-producing *Enterobacteriaceae* (CPE), MBL-producing bacteria only represent 5% in Portugal (Manageiro et al., 2018; Gorgulho et al., 2020).

IMP-22 was first described in Italy in two non-related environmental strains of *Pseudomonas fluorescens* as well as in one clinical isolate of *Pseudomonas aeruginosa* (Pellegrini et al., 2009). Since the first description, the same enzyme was then described also in a *Pseudomonas* spp. single clinical isolate from Austria (Duljasz et al., 2009) and recently emerged in Spain, always in the clinics, associated first with *P. aeruginosa* (Viedma et al., 2012) and then mainly with *K. pneumoniae* (Miro et al., 2013; Pena et al., 2014) but also with *E. coli* (Ortega et al., 2016).

Here, we report and describe the successful installation of IMP-22-producing *K. pneumoniae* in a Portuguese acute care hospital (in the North of Portugal), due to both vertical and horizontal dissemination. Furthermore, we describe for the first time the breach of the hospital frontier, with the detection and characterization of an IMP-22-producing *K. pneumoniae* isolate, *via* the screening of intestinal colonizers of residents of a long-term care facility (LTCF). These results ultimately highlight the circulation of patients between hospital and extra-hospital care settings as the most probable justification for the “dissemination of multiresistant bacteria toward the community.”

## Materials and Methods

### Hospital Settings and Clinical Carbapenem-Resistant *K. pneumoniae* Isolates

This study was performed in the context of one of the largest acute care hospitals in the North of Portugal (705 beds), covering a population of 1.2 million people (hereafter called Hospital A). During a one-year study period (from March 2011 to May 2012) *K. pneumoniae* clinical isolates showing reduced susceptibility to carbapenems (imipenem or ertapenem or meropenem) were identified as part of routine diagnostics in the hospital Clinical Pathology Service. Isolates were collected from both inpatients admitted to the internal medicine service and from patients admitted to the hospital emergency ward.

### Long-Term Care Facility and Carbapenem-Resistant *K. pneumoniae* Intestinal Colonizers

An extra-hospital healthcare institution for dependent and old people in the North of Portugal was studied. The LTCF with 54 beds has three different typologies of care: long-term maintenance (LTM, 22 beds), medium-term and rehabilitation (MTR, 22 beds), and palliative care (PC, 10 beds). The institution is located in the same geographic area as Hospital A (distance of 4 Km), and consequently, the circulation of patients between these two healthcare institutions occurs frequently.

Thirty-eight fecal samples from LTCF residents were collected between January and February 2012, suspended in Brain Heart Infusion (BHI; Oxoid, Hampshire, United Kingdom), and incubated overnight at 37°C. The enriched suspensions were then plated onto MacConkey agar plates (Oxoid, Hampshire, United Kingdom) supplemented with meropenem (1mg/l). Isolates that grew in the selective media were re-inoculated in a new plate to exclude any satellite growers (maximum of four random colonies per plate).

### Bacterial Identification and Antimicrobial Susceptibility Determination

The clinical isolates were identified using the Vitek^®^ 2 automated system (bioMérieux, Marcy l’Étoile, France). Bacteria isolated as part of the intestinal colonization screening were identified using the bacterial identification biochemical galleries API^®^ 20E and ID^®^32GN (bioMérieux).

The antimicrobial susceptibility of clinical isolates was assessed through the determination of the minimum inhibitory concentration (MIC) of different antimicrobial agents, performed using the Vitek^®^ 2 (bioMérieux) and/or WalkAway (Beckman Coulter, Brea, CA, United States) automated systems. The MICs detected for ampicillin, piperacillin, ticarcillin, amoxicillin + clavulanic acid, piperacillin + tazobactam, ticarcillin + clavulanic acid, cephalothin, cefuroxime, ceftazidime, cefotaxime, cefepime, aztreonam, imipenem, ertapenem, meropenem (β-lactams), gentamicin, tobramycin, amikacin, minocycline, ciprofloxacin, levofloxacin, pefloxacin, nitrofurantoin, trimethoprim + sulfamethoxazole, and rifampicin (non-β-lactams) were interpreted into susceptible, intermediate susceptible or resistant according to the clinical and laboratory standards institute (CLSI) guidelines (Queenan and Bush, 2007; Supplementary Table S1). For isolates collected in the LTCF intestinal colonization screening, antimicrobial susceptibility was determined by disk-diffusion methods; susceptibility to both β-lactams [ampicillin (10μg), amoxicillin + clavulanic acid (20+10μg), ceftazidime (30μg), cefotaxime (30μg), cefepime (30μg), cefoxitin (30μg), aztreonam (30μg), imipenem (10μg), ertapenem (10μg), and meropenem (10μg)] and non-β-lactam antibiotics [streptomycin (10μg), gentamicin (10μg), netilmicin (30μg), tobramycin (10μg), amikacin (30μg), tetracycline (30μg), nalidixic acid (30μg), ciprofloxacin (5μg), nitrofurantoin (300μg), chloramphenicol (30μg), tigecycline (15μg), and trimethoprim + sulfamethoxazole (1.25/23.75μg)] was defined according to the CLSI guidelines (CLSI, 2013) or the EUCAST criteria in the case of tigecycline^1^ (Supplementary Table S2).

### Carbapenemases Phenotypic Screening

An initial carbapenemase production screening (MBLs) was performed using the double disk synergism method (DDSM) - IMP (10μg) versus IMP (10μg)+EDTA (0,5M), followed by the confirmatory MBL *E*-test IP/IPI [(MIC determination; IMP (4-256μg/ml) versus IMP (1-364μg/ml)+EDTA (constant level)] (bioMérieux, Marcy l’Étoile, France). The *E*-test was considered MBL suggestive when the MIC ratio of imipenem/imipenem plus EDTA was ≥8 and/or when the presence of a phantom zone or deformation of the inhibitory ellipse was observed. The modified hodge test (MHT) was performed in parallel, to screen for non-MBL carbapenemase production. Briefly, an imipenem disk (10μg) was placed at the center of a Müeller-Hinton agar plate (Oxoid, Hampshire, United Kingdom), previously inoculated with *E. coli* ATCC 25922, and the clinical isolates were streaked heavily from the edge of the disk toward the edge of the plate. The MHT was considered positive when *E. coli* growth was observed within the usual inhibition zone of the imipenem disk (CLSI, 2013). As a final confirmatory step the biochemical Blue-Carba test was performed as described elsewhere (Pires et al., 2013).

### Characterization of Antibiotic Resistance Genes

Total DNA was extracted from all isolates *via* the boiling of single bacterial colony suspensions for 10min, followed by a 5min centrifugation step at 15,000rpm. The supernatant was then collected and stored at 4°C until further use. Relevant beta-lactamase [*bla*_TEM_, *bla*_OXA,_ *bla*_SHV_ (Dallenne et al., 2010)_,_ and *bla*_CTX-M group 1_ (Machado et al., 2005)] and carbapenemase [*bla*_VIM_, *bla*_IMP_, *bla*_KPC_, *bla*_OXA-48_, and *bla*_NDM_ (Poirel et al., 2011)] genes were screened using the primers and amplification conditions described in the literature (Machado et al., 2005; Dallenne et al., 2010; Poirel et al., 2011; Goncalves et al., 2016; Teixeira et al., 2016). Whenever relevant, amplicons were sequenced using the ABI-PRISM 3100 automatic genetic analyzer (Thermo Fischer Scientific, Waltham, MA, United States). Sequence analysis and alignment were performed using the National Center for Biotechnology Information tool.^2^ As a final confirmation step, IMP-22 specific primers were used (Pellegrini et al., 2009). A compilation of all primer sequences used in this study can be found in (Goncalves et al., 2016).

### Determination of the Clonal Relationships *via* Pulsed-Field Gel Electrophoresis

The clonal relationships of the *K. pneumoniae* clinical isolates were studied *via* pulsed-field gel electrophoresis (PFGE), after total genomic DNA digestion with *XbaI* (Gautom, 1997). Briefly, carbapenem-resistant clinical isolates were cultured in brain heart infusion (BHI) for 24h at 37°C, then “trapped” into 1.6% agarose plugs. A lysis step was performed at 54°C for 2h (50mm Tris, 50mm EDTA, 1% N-lauryl-sarcosine, 0.1 mg/ml proteinase K, pH 8.0), followed by 2–3 washing cycles, and afterward, the digestion overnight with 30U of *XbaI* at 37°C. Total DNA digests were separated on 1.0% agarose gels (SeaKem Gold Agarose, Lonza, Basel, Switzerland) *via* PFGE using the CHEFF DR III system (Bio-Rad Laboratories, Hercules, CA, United States) and the following conditions: electric field strength of 6V/cm^2^ (200V), 14°C, and pulse time of 15s–25s for 16h. After electrophoresis, the gels were stained with ethidium bromide (10μg/ml) for 30min and watched under a UV light (Bio-Rad Laboratories). Data analysis was performed using the BIONUMERICS software, version 8.0 (bioMérieux, Marcy l’Étoile, France); the UPGMA algorithm based on the Dice coefficient (1.0% band tolerance; 1.0% optimization) was applied. The PFGE profiles were defined on the basis of DNA banding patterns in accordance with the criteria defined by Tenover et al. (1995). Isolates with a pattern similarity profile above ≥80% were considered identical.

### Horizontal Gene Transfer Assessment

Conjugation experiments were performed to investigate the transfer of carbapenem resistance determinants. *E. coli* HB101 (azide resistant, lactose-negative) was used as the recipient strain. Donor and recipient bacterial strains were individually grown overnight in Trypticase Soy Broth (TSB; Oxoid, Hampshire, United Kingdom) and drops of donor and recipient bacterial suspensions were then mixed on the surface of a Müeller-Hinton Agar plate (Oxoid) and re-incubated at 37°C for 24h. The resulting bacterial growth was re-inoculated on Müeller-Hinton medium supplemented with meropenem or ceftazidime (10mg/l) and azide (100μg/ml) and incubated for a maximum of 72h at 37°C. Growing colonies on the selective medium were randomly chosen and inoculated in MacConkey agar (Oxoid) to assess lactose fermentation. Lactose non-fermenters were subjected to antimicrobial susceptibility determination and to genotypic characterization as above stated.

### Ethics Statement

This research was conducted in accordance with the Declaration of Helsinki Ethical Principles. This study was approved by the Ethics Committee of Hospital de Braga, Braga, Portugal. Additionally, human fecal sample collection was performed in accordance with the Good Clinical Practice guidelines; the LTCF direction provided the necessary authorization to conduct this study. Of note, all of the study participants provided written informed consent.

## Results

### Hospital *K. pneumoniae* Isolates: Clinical Context

Eight carbapenem-resistant *K. pneumoniae* showing reduced susceptibility to at least one of the carbapenems tested were isolated from different biological samples of seven distinct hospitalized patients: five inpatients of the internal medicine service of Hospital A and two patients admitted to the emergency ward of the same hospital. The most common type of biological sample from which these bacteria were isolated was sputum (*n*=5), followed by urine (*n*=2) and blood (*n*=1; Table 1). Two of the carbapenem-resistant *K. pneumoniae* (isolates H13 and H50) were isolated from the same patient (patient 3) in different periods (December 2011 and April 2012, respectively; Table 1). The patients were mostly elderly (median age 74years; range 36–92years) with distinct underlying illnesses, namely urinary tract infection (*n*=1), endocarditis (*n*=1), renal insufficiency (*n*=2), brain tumor (*n*=1), nosocomial pneumonia (*n*=1), acute pancreatitis (*n*=1), respiratory insufficiency (*n*=2), and bladder tumor (*n*=1; Table 1). All of the patients had previous hospitalization history in Hospital A, with many of them spending prolonged periods at the internal medicine ward. Of note, patients 3, 6, and 7 received meropenem therapy during their hospitalization period (Table 1).

**Table 1:** Hospital *K. pneumoniae* isolates: clinical context.

Patient Nr.	Date (month/year)	Isolate ID	Age/Sex	Hospital service	Biological products	Underlying diseases	Origin^a^	Treated with meropenem during hospital admission
1	March 2011	H7	92/F	Internal medicine	Blood culture	Urinary tract infection	Domicile	No
2	May 2011	H8	36/M	Internal medicine	Urine	Endocarditis	No residence identified	No
3	December 2011 (1st isolate)	H13	86/M	Internal medicine	Urine	Renal insufficiency	Domicile/LTCF	Yes
	April 2012 (2nd isolate)	H50		Internal medicine	Sputum			
4	December 2011	H15	77/F	Internal medicine	Sputum	Brain tumor and nosocomial pneumonia	Hospital B	No
5	January 2012	H40	80/M	Emergency ward	Sputum	Acute pancreatitis and respiratory insufficiency	Domicile	No
6	March 2012	H41	89/M	Emergency ward	Sputum	Renal and respiratory insufficiency	LTCF	Yes
7	May 2012	H52	56/M	Internal medicine	Sputum	Bladder tumor	Hospital C	Yes

*F, female; M, male*.

a *origin of the patient before admission into Hospital A, hospitals B and C are different, but in the same geographic area of Hospital A*.

### LTCF Carbapenem-Resistant *K. pneumoniae* Isolates: Contextualization

Four carbapenem-resistant *K. pneumoniae* isolates (10.53%, 4/38) with reduced susceptibility to imipenem were detected in fecal samples of residents of a LTCF (two different typologies of care; LTM, *n*=3; MTR, *n*=1; Table 2). The four residents colonized with carbapenem-resistant bacteria were mostly elderly (median age 72.5years; range 63–82years), with previous history of stroke (*n*=2), stroke associated with other pathologies (endocarditis and pneumonia; *n*=1), and chronic renal insufficiency (*n*=1; Table 2). Three of them had recent hospitalization history in Hospital A: two spent prolonged periods in the internal medicine ward (residents A and D) while the third one was admitted to the orthopedics service (resident B). The fourth resident (C) had hospitalization history in three different hospitals in the same geographic area of Hospital A (one hospital in Braga district and two hospitals in Porto district; Table 2).

**Table 2:** Extra-hospital carbapenem-resistant *K. pneumoniae* isolates: epidemiological contextualization.

LTCF resident code	Date (month/year)	Isolate ID	Age/Sex	LTCF typology	Underlying diseases	Resident origin^a^
A	February 2012	22	63/M	MTR	Stroke	Hospital A – IM
B	February 2012	34	80/F	LTM	Stroke	Hospital A – O
C	February 2012	31	65/F	LTM	Stroke, endocarditis pneumonia	Hospitals B, C and D^*^
D	February 2012	24	82/F	LTM	Chronic renal insufficiency	Hospital A – IM

*F, female; IM, internal medicine ward; LTM, long-term maintenance; LTCF, long-term care facility; M, male; MTR, medium-term and rehabilitation; O, orthopedics service*.

a *origin of the resident before LTCF admission.*

* *hospitalization, in chronological order, in three different hospitals: hospital B - Braga district; hospitals C and D, Porto district*.

### Antimicrobial Susceptibility Patterns of the Carbapenem-Resistant *K. pneumoniae* Isolates

All clinical isolates showed reduced susceptibility to meropenem (MIC ≥16; R). Additionally, of the set of clinical isolates analyzed, six presented resistance to Ertapenem, and two to imipenem (three others showed an intermediate phenotype; Table 3). Importantly, most of the clinical isolates also showed resistance to expanded-spectrum cephalosporins, other *β*-lactams and β-lactam/β-lactamase inhibitor combinations; additionally resistance to gentamycin (*n*=2), tobramycin (*n*=5), ciprofloxacin (*n*=6), norfloxacin (*n*=1), pefloxacin (*n*=1), trimethoprim/sulfamethoxazole (*n*=7), and tetracycline (*n*=6) was also detected (Table 3).

**Table 3:** IMP-22-producing *K. pneumoniae* clinical isolates: phenotypic and genotypic antimicrobial susceptibility patterns.

Patient Nr.	Isolate ID	MICs of carbapenem antibiotics (mg/l)			MICs of other β-lactam antibiotics (mg/l)								Resistance to non-β-lactam antibiotics^a^	MBL E-test	Modified Hodge test	Blue-Carba test	Resistance determinants
		IPM	ETP	MEM	AMC	AMP	P/T	CAZ	CTX	FEP	ATM	FOX					
1	H7	2 I	4 R	≥16 R	≥32 R	≥32 R	≥32 I	≥64 R	8 R	–	≤1S	8 R	TE, T/S	Negative	Negative	Positive	*bla_IMP-22_*
2	H8	≤1S	≥8 R	≥16 R	>16/8 R	>16 R	8S	≥64 R	>32 R	16 I	≤1S	>16 R	TE, CIP, GM, TOB, PE, T/S	Negative	Negative	Positive	*bla_IMP-22_*
3	H13	≤1S	≤1S/I	≥16 R	16/8 I	>16 R	≤8S	≥16 R	≥16 R	8S	–	≥16 R	TE, CIP, NOR, T/S	Negative	Negative	Positive	*bla_IMP-22_*
	H50	4 R	>1 R	≥16 R	≥32 R	≥32 R	64 I	≥64 R	16 R	–	–	–	CIP, TOB	Negative	Negative	Positive	*bla_IMP-22_*
4	H15	4 R	4 R	≥16 R	>16/8 R	>16 R	≤8S	>16 R	>16 R	8S	–	>32 R	TE, CIP, TOB, T/S	Negative	Negative	Positive	*bla_IMP-22_*
5	H40	2 I	4 R	≥16 R	≥32 R	>16 R	8S	≥64 R	≥64 R	16 I	≤1S	≥64 R	TE, CIP, TOB, T/S	Negative	Negative	Positive	*bla_IMP-22_*
6	H41	2 I	>1 R	≥16 R	>16/8 R	>16 R	≤8S	>16 R	>32 R	8S	–	>16 R	TE, CIP GM, TOB, T/S	Negative	Negative	Positive	*bla_IMP-22_*
7	H52	≤1S	≤1S/I	≥16 R	16/8 I	16 I	≤4S	16 I	>16 R	≤1S	–	>16 R	T/S	Negative	Negative	Positive	*bla_IMP-22_*

*IPM, imipenem; ETP, ertapenem; MEM, meropenem; AMC, amoxicillin+clavulanic acid; AMP, ampicillin; P/T, piperacillin+tazobactam; CAZ, ceftazidime; CTX, cefotaxime; FEP, cefepime; ATM, aztreonam; FOX, cefoxitin; TE, tetracycline; CIP, ciprofloxacin; GM, gentamicin; TOB, tobramycin; PE, pefloxacin; T/S, trimethoprim+sulfamethoxazole; NOR, norfloxacin. (a) - antibiotic resistance to non-β-lactamic antibiotics transferred by conjugation is underlined, (−) not evaluated*.

The four intestinal colonization *K. pneumoniae* isolates also showed reduced susceptibility to imipenem (Table 4). Two of them also showed reduced susceptibility to ertapenem (isolates 22 and 24), with only one (isolate 22) showing resistance to the three carbapenems tested (imipenem, ertapenem, and meropenem). The majority of the intestinal isolates also showed resistance to expanded-spectrum cephalosporins (*n*=3), other *β*-lactams (*n*=4), β-lactam/β-lactamase inhibitor combinations (*n*=4) as well as to non-β-lactam antibiotics, including tetracycline (*n*=3), trimethoprim/sulfamethoxazole (*n*=3), nalidixic acid (*n*=4), ciprofloxacin (*n*=4), chloramphenicol (*n*=2), and streptomycin (*n*=1; Table 4).

**Table 4:** Carbapenem-resistant intestinal colonization *K. pneumoniae* isolates: phenotypic and genotypic antimicrobial susceptibility features.

LTCF resident code	Isolate ID	Resistance to carbapenems	Resistance to non-β-lactam antibiotics	Blue-Carba test	Resistance determinants
A	22	IPM, ETP, MEM	TE, CIP, T/S, S, NA	Positive	*bla_IMP-22_*
B	34	IPM	CIP, T/S, NA, C	Negative	–
C	31	IPM	TE, CIP, NA	Negative	–
D	24	IPM, ETP	TE, CIP, T/S, NA, C	Negative	–

*LTCF, long-term care facility; IPM, imipenem; ETP, ertapenem; MEM, meropenem; TE, tetracycline; CIP, ciprofloxacin; T/S, trimethoprim+sulfamethoxazole; S, streptomycin; NA, nalidixic acid; C, chloramphenicol; (−) negative*.

Of note, all clinical and intestinal colonization *K. pneumoniae* isolates were defined as multidrug-resistant (MDR) in accordance with the definition proposed by Magiorakos and colleagues (non-susceptible to ≥1 agent in ≥3 antimicrobial categories; Magiorakos et al., 2012).

### Characterization of the Carbapenem Resistance Mechanisms

Interestingly, the production of MBL in the context of all of the *K. pneumoniae* clinical isolates was initially defined as negative, as per the MBL *E*-test IP/IPI. Additionally, the results of the MHT were also negative for all of the clinical isolates. However, contrary to these findings, the results of the Blue-Carba test (all positive), suggested the expression of carbapenemases in all clinical isolates. Similarly, one of the intestinal colonization *K. pneumoniae* isolates was also determined as a carbapenemase producer, as per the results of the Blue-Carba test. Of note, according to the CLSI guidelines, extended-spectrum β-lactamase production was not detected, both in the clinical and intestinal colonization carbapenem-resistant isolates.

Finally, the *bla*_IMP-22_ gene was detected *via* PCR followed by sequencing in all of the eight *K. pneumoniae* clinical isolates and in one of the carbapenem-resistant intestinal isolates, part of the commensal intestinal microbiota of one LTCF resident (isolate 22). PCR amplification was further confirmed using IMP-22 specific primers, supporting our sequencing results. No other carbapenemase genes were detected in the set of *K. pneumoniae* isolates.

### Clonal Relationships of the IMP-22-Producing *K. pneumoniae* Isolates

**Figure 1:**
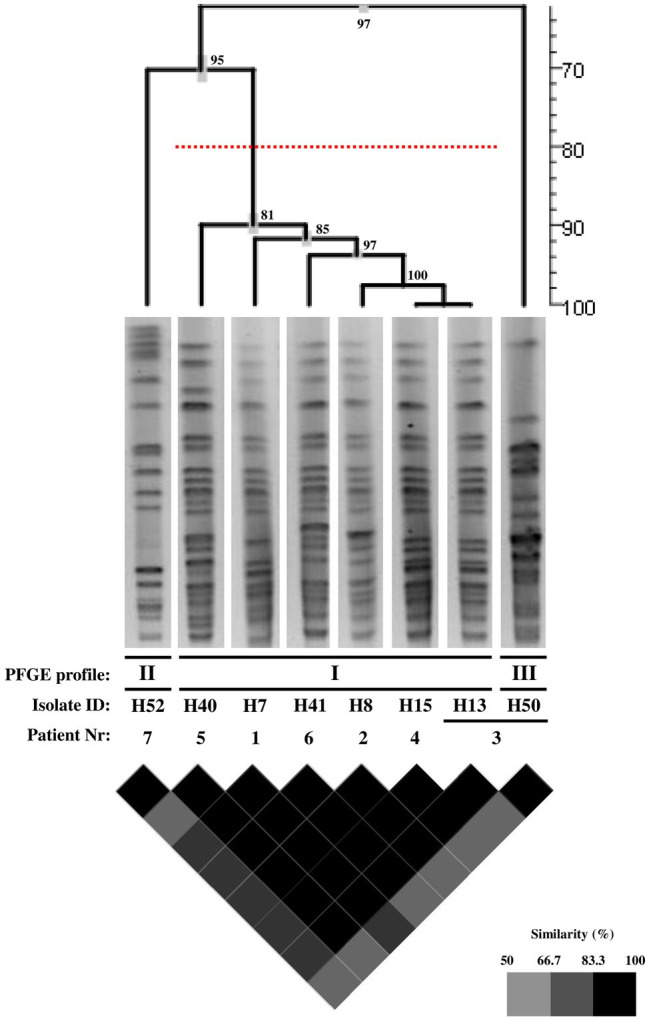
PFGE analysis of the IMP-22-producing *K. pneumoniae* clinical isolates. The clonal relationships between the eight *K. pneumoniae* clinical isolates were studied *via* pulsed-field gel electrophoresis (PFGE), after total genomic DNA digestion with *XbaI*. Cluster analysis was performed using the UPGMA algorithm based on the Dice coefficient (1.0% band tolerance; 1.0% optimization). The dendrogram obtained is shown, as are the isolated band patterns’ used in the cluster analysis. Additionally, a similarity matrix is also provided. Together with both the dendrogram and the similarity matrix, a similarity scale (in percentage) is provided (distance or color code, respectively). Additionally, in the dendrogram, the cophenetic correlation values are given in each node, as is the standard deviation (in grey). The PFGE profiles were defined on the basis of DNA banding patterns in accordance with the criteria defined by Tenover et al. (1995); isolates with a pattern similarity profile above ≥80% (represented by the dashed red line in the dendrogram) were considered identical.

For epidemiological purposes, since many different carbapenem-resistant isolates were isolated in a short period of time in Hospital A, next, we investigated the genetic relationships of the eight IMP-22-producing *K. pneumoniae* clinical isolates using PFGE. Three distinct PFGE profiles (I to III) were revealed. Importantly, six of the clinical isolates (namely H7, H8, H13, H15, H40, and H41) shared the same profile (profile I), indicating that these isolates are genetically identical and have the same origin (Figure 1). The two remaining isolates, H52 and H50 showed two different profiles (profiles II and III, respectively; Figure 1). Of note, the isolates belonging to the single major clone (clone I) were isolated from four patients admitted to the medicine ward (isolates H7, H8, H13, and H15), and two patients admitted to the emergency ward (isolates H40 and H41); however, these two patients had previously been admitted to the same hospital. Interestingly, the two isolates collected from the same patient showed different PFGE patterns: H13, with the predominant profile I, was isolated during a first prolonged stay in Hospital A, while H50, with a unique typing pattern unrelated to profile I, was isolated during a second hospitalization, after a period spent in an LTCF in the same geographic region of Hospital A (the same LTCF from which the IMP-22-producing *K. pneumoniae* intestinal colonizer was isolated).

### IMP-22-Producing *K. pneumoniae* Isolates of the Dominant Clone Are Effectively Able to Horizontally Transfer the Carbapenem Resistance Determinant

Since the findings in the context of the patient from which two isolates were collected suggest the horizontal transfer of *bla*_IMP-22_ (two different PFGE types), we further performed conjugation experiments. Importantly, our results confirmed the above hypothesis; we observed the transference not only of the carbapenem resistance determinant but also of resistance determinants to non-β-lactam antibiotics (Table 3; underlined), in the context of six clinical isolates (H7, H8, H13, H15, H40, and H41), all belonging to the single major clone. On the other hand, no conjugation was achieved in the context of the two remaining clones isolated from clinical samples, as well as of the carbapenem-resistant *K. pneumoniae* intestinal isolates. Importantly, the presence of the *bla*_IMP-22_ gene in all of the trans-conjugants obtained was confirmed *via* PCR and sequencing.

## Discussion

Since the widespread use of carbapenems in the clinical settings, carbapenem-resistant *Enterobacteriaceae* have been increasingly detected worldwide (including in Portugal), not only in hospitals, but also in extra-hospital healthcare institutions, as well as in the environment (Logan and Weinstein, 2017). Among these bacteria, *K. pneumoniae* are formidable nosocomial pathogens with the potential to acquire resistance to multiple antimicrobial agents and consequently associated with high mortality and morbidity; of note, the emergence of MDR *K. pneumoniae* in extra-hospital healthcare institutions in the community, including LTCF, has been more and more reported (Navon-Venezia et al., 2017). Here, we describe for the first time not only the emergence of IMP-22-producing *K. pneumoniae* in an acute care hospital in the North of Portugal, but also, and more importantly, the breach of the hospital frontier toward the community, with the detection of one IMP-22-producing *K. pneumoniae* isolate as a component of the fecal microbiota of a resident of an extra-hospital health care setting (LTCF) in the same geographic area.

IMP-22 MBLs, first described associated with *Pseudomonas* spp. in different European countries are now emerging in *K. pneumoniae* in the Iberian Peninsula. In fact, the finding of the *bla*_IMP-22_ gene in *P. fluorescens* environmental strains (Pellegrini et al., 2009) as well as in *P. aeruginosa*, *K. pneumoniae,* and *E. coli* (Duljasz et al., 2009; Pellegrini et al., 2009; Viedma et al., 2012; Miro et al., 2013; Pena et al., 2014; Ortega et al., 2016) clinical isolates in different European countries suggest the ongoing spread of this *bla*_MBL_ gene among Gram-negative bacteria. Importantly, our results support this notion and highlight the spread of this particular resistance determinant *via* both vertical and horizontal transmission, not only in the clinics but also in the community. Indeed, our results show a clonal spread of IMP-22-producing *K. pneumoniae* in the clinical settings, but also the possible plasmid-mediated spread of the *bla*_IMP-22_ gene in both the clinics and the community. These data reflect the complexity of the spread of CPE alerting for the need for adequate infection control practices in all healthcare institutions.

Different clonal outbreaks caused by carbapenem-resistant *K. pneumoniae* have been reported in particular hospitals in Portugal; however, most if not all of the reported outbreaks were associated with non-metallo-carbapenemases (including KPC-3 and OXA-48; (Conceicao et al., 2005; Rodrigues et al., 2014; Vubil et al., 2017; Manageiro et al., 2018; Mendes et al., 2018; Aires-de-Sousa et al., 2019; Perdigao et al., 2019; Gorgulho et al., 2020; Guerra et al., 2020; Lopes et al., 2020). In fact, only around 5% of the reported CPE in Portugal are associated with metallo-carbapenemases (Manageiro et al., 2018; Gorgulho et al., 2020). Therefore, our report of an outbreak caused by IMP-22-producing *K. pneumoniae* has epidemiological relevance, complementing the landscape of carbapenemase-producing bacteria in Portugal. Of note, the first IMP-22-producing *K. pneumoniae* clinical isolate was detected in March 2011, and since then, during one-year period (till May 2012) seven more isolates were found in the same hospital (and one more in the community). These data may suggest the successful installation of such resistant bacteria in the North of Portugal, with public health implications.

The original source and potential route of transmission of these IMP-22-producing *K. pneumoniae* isolates (clinical and intestinal commensal) is not known. However, our results suggest a common source, at least considering the IMP-22-producing *K. pneumoniae* clinical isolates of the predominant clone. The link between most patients admitted to Hospital A was their stay in the medicine ward; therefore, it is not unreasonable to speculate that transmission occurred during the hospital stay. Of note, although the *K. pneumoniae* isolates from patients 5 and 6 were detected in the emergency ward, both patients were previously admitted for a long period to the medicine ward of Hospital A; after hospital discharge, patient 5 went home, and patient 6 went to an LTCF in the same geographic region (the facility where the IMP-22 positive intestinal colonizer strain was isolated), but then returned to Hospital A due to health status complications. Therefore, intestinal colonization of these patients with IMP-22-producing *K. pneumoniae* is a serious hypothesis to be considered, after hospital discharge. Importantly, our results are in line with the more and more recognized notion that extra-hospital care institutions are a highway for the escape of MDR bacteria from the hospitals to the community, as well as for the (re)-introduction of MDR bacteria into hospitals (Masgala et al., 2015; Mody et al., 2018).

Interestingly, our results also suggest that, although most transmission events were clonal, some of them were horizontal in nature. This was particularly clear in the context of patient number 3, with two different isolates (detected during two distinct hospitalizations) showing non-related PFGE profiles. This, together with the fact that we were able to obtain trans-conjugants with all of the clinical isolates from the predominant clone suggest that these *K. pneumoniae* isolates are able to disseminate this particular carbapenem resistance determinant. Our results are, therefore, worrisome, thinking on the possibility of the emergence of more fit IMP-22-producing *Enterobacteriaceae* and their installation in the clinics and the community, in Portugal and even abroad (depending on the dissemination success of the bacteria).

Remarkably, MBL detection, as per the *E*-test IP/IPI, was negative in the context of all IMP-22-producing isolates, highlighting the need for the use of adequate phenotypic approaches to detect these particular carbapenem-resistant strains. Although according to some reports there is still “no gold standard CPE detection method” (Berry et al., 2019), many recognize the genotypic approach (detection of carbapenemase-encoding genes) as the most suitable (Nordmann and Poirel, 2013). However, the diagnosis capacity is not homogeneous around the world; the COVID-19 pandemic exposed the clear inequality-derived differences among countries (Giri and Rana, 2020; Millar, 2020). Therefore, phenotypic methods are still widely used as a primary approach to detect CPE. Importantly, our results highlight the need to use complementary (phenotypic) methods, to prevent the potential disregard of carbapenemase-producing strains, such as the IMP-22-producing *K. pneumoniae* isolates we report here; metallo-carbapenemase producers not detected using the standard *E*-test IP/IPI method (thus inadequate for the detection of IMP-22-producing *K. pneumoniae*), but detected using the Blue-Carba test. Of note, if possible, the genotypic determination of MBL is recommended in situations of reduced susceptibility to carbapenems, excluding imipenem. Altogether, our results alert for the need for the correct detection of CPE in routine clinical microbiology testing, to avoid outbreak installation.

The early identification of CPE in hospitalized patients and the implementation of adequate infection control measures are, thus, extremely important to prevent the persistence and spread of carbapenem-resistant bacteria (Magiorakos et al., 2017), such as the IMP-22-producing *K. pneumoniae* strains we report in this study, not only in the hospital settings but also in the community. In fact, after hospital discharge, patients can remain colonized and contribute for the dissemination of these MDR *K. pneumoniae* within extra-hospital care settings, namely, LTCF, and nursing homes (Chen et al., 2021). Therefore, the early identification of carriers and the implementation of adequate control strategies are essential to prevent nosocomial outbreaks. This is precisely what we show in this study. The individual colonized with the IMP-22-producing *K. pneumoniae* had previous hospitalization history in the medicine ward of the same acute care hospital where the clinical isolates were detected; therefore, our results suggest that this individual was colonized during hospitalization and served as a silent vehicle transporting the IMP-22-producing *K. pneumoniae* toward the community after discharge.

This study is not without limitations. First, antibiotic susceptibility of clinical and community isolates was assessed using two different methods, MIC determination, and disc-diffusion assays, respectively. However, for both methods, the CLSI/EUCAST guidelines were strictly followed. Second, the community isolate was not included in the analysis of clonal relationships. Therefore, we do not know whether this isolate belongs to the major clone, to one of the two single clones, or if it is a different clone; all of these options are possible. Third, although our results clearly suggest that the single clones were derived from horizontal dissemination events, this must yet be clearly shown. To address these limitations, we plan, in a follow-up study, to perform whole-genome sequencing of the isolates that will allow us to determine their MLST (and the clonal relationships of all of the isolates) and their resistome, as well as to perform detailed plasmid analyses and undoubtedly prove the horizontal transfer of the IMP-22 resistance determinant.

Altogether, our results align with the dogma that the presence of patients colonized with MDR *Enterobacteriaceae* in LTCF can represent a serious risk of dissemination and potential infection of elderly patients in the community, requiring, therefore strict epidemiological attention. In the future, as a preventive measure of the dissemination of multidrug-resistant bacteria, we suggest the active screening of intestinal colonization, both at hospital admission and hospital discharge, as well as, sporadically, in extra-hospital healthcare settings including LTCF and nursing homes; the detection of carbapenem-resistant bacteria at these stages will allow the implementation of rational infection control measures, with the potential to prevent outbreaks both in the clinics and the community.

## Data Availability Statement

The raw data supporting the conclusions of this article will be made available by the authors, without undue reservation.

## Ethics Statement

This research was conducted in accordance with the Declaration of Helsinki Ethical Principles. This study was approved by the Ethics Committee of Hospital de Braga, Braga, Portugal. Additionally, human fecal sample collection was performed in accordance with the Good Clinical Practice guidelines; the LTCF direction provided the necessary authorization to conduct this study. Of note, all of the study participants provided written informed consent.

## Author Contributions

DG and HF conceived and designed the experiments. DG and PC performed the experiments and wrote the original draft. DG, PC, and HF analyzed the data. DG, PC, AF, CI, FB, AE, and HF critically discussed the results and critically revised and edited the paper. HF assured the funding and contributed with the reagents, materials, and analysis tools. AF, CI, FB, and AE provided the clinical strains. All authors approved the current version of this manuscript.

## Funding

This study was supported by internal funding.

## Conflict of Interest

The authors declare that the research was conducted in the absence of any commercial or financial relationships that could be construed as a potential conflict of interest.

## Publisher’s Note

All claims expressed in this article are solely those of the authors and do not necessarily represent those of their affiliated organizations, or those of the publisher, the editors and the reviewers. Any product that may be evaluated in this article, or claim that may be made by its manufacturer, is not guaranteed or endorsed by the publisher.

## Footnotes

^1^ http://www.eucast.org/clinicalbreakpoints/

^2^ http://blast.ncbi.nlm.nih.gov/Blast.cgi

## Supplementary Material

The Supplementary Material for this article can be found online at:

https://www.frontiersin.org/articles/10.3389/fmicb.2021.777054/full#supplementary-material

## References

[ref1] Aires-de-Sousa M. Ortiz de la Rosa J. M. Goncalves M. L. Pereira A. L. Nordmann P. Poirel L. (2019). Epidemiology of Carbapenemase-producing Klebsiella pneumoniae in a hospital, Portugal. Emerg. Infect. Dis. 25:1632. doi: 10.3201/eid2509.190656, PMID: 31441424 PMC6711212

[ref2] Berry C. Davies K. Woodford N. Wilcox M. Chilton C. (2019). Survey of screening methods, rates and policies for the detection of carbapenemase-producing Enterobacteriaceae in English hospitals. J. Hosp. Infect. 101, 158–162. doi: 10.1016/j.jhin.2018.08.005, PMID: 30092291

[ref3] CDC (2019) Infographic: Antibiotic Resistance The Global Threat Available at: https://www.cdc.gov/globalhealth/infographics/antibiotic-resistance/antibiotic_resistance_global_threat.htm. (Accessed May 27, 2021).

[ref4] Chen H. Y. Jean S. S. Lee Y. L. Lu M. C. Ko W. C. Liu P. Y.. (2021). Carbapenem-resistant Enterobacterales in long-term care facilities: A global and narrative review. Front. Cell. Infect. Microbiol. 11:601968. doi: 10.3389/fcimb.2021.601968, PMID: 33968793 PMC8102866

[ref5] CLSI (2013). Performance standards for antimicrobial susceptibility testing. Wayne, PA: Clinical and Laboratory Standards Institute. Supplement M100–S23 p.

[ref6] Conceicao T. Brizio A. Duarte A. Barros R. (2005). First isolation of Bla(VIM-2) in *Klebsiella oxytoca* clinical isolates from Portugal. Antimicrob. Agents Chemother. 49:476. doi: 10.1128/AAC.49.1.476.2005, PMID: 15616343 PMC538894

[ref7] Dallenne C. Da Costa A. Decre D. Favier C. Arlet G. (2010). Development of a set of multiplex PCR assays for the detection of genes encoding important beta-lactamases in Enterobacteriaceae. J. Antimicrob. Chemother. 65, 490–495. doi: 10.1093/jac/dkp498, PMID: 20071363

[ref8] Duljasz W. Gniadkowski M. Sitter S. Wojna A. Jebelean C. (2009). First organisms with acquired metallo-beta-lactamases (IMP-13, IMP-22, and VIM-2) reported in Austria. Antimicrob. Agents Chemother. 53, 2221–2222. doi: 10.1128/AAC.01573-08, PMID: 19223629 PMC2681493

[ref9] Gautom R. K. (1997). Rapid pulsed-field gel electrophoresis protocol for typing of *Escherichia coli* O157:H7 and other gram-negative organisms in 1 day. J. Clin. Microbiol. 35, 2977–2980. doi: 10.1128/jcm.35.11.2977-2980.1997, PMID: 9350772 PMC230100

[ref10] Giri A. K. Rana D. R. S. J. B. (2020). Charting the challenges behind the testing of COVID-19 in developing countries: Nepal as a case study. Biosafety Health 2:53. doi: 10.1016/j.bsheal.2020.05.002 PMC7219426 38620322

[ref11] Goncalves D. Cecilio P. Ferreira H. (2016). Nursing homes and long-term care facilities: reservoirs of CTX-M-15-producing *Escherichia coli* O25b-ST131 in Portugal. J. Glob. Antimicrob. Resist. 7, 69–71. doi: 10.1016/j.jgar.2016.08.001, PMID: 27665185

[ref12] Gorgulho A. Grilo A. M. de Figueiredo M. Selada J. (2020). Carbapenemase-producing Enterobacteriaceae in a Portuguese hospital - a five-year retrospective study. Germs 10, 95–103. doi: 10.18683/germs.2020.1190, PMID: 32656106 PMC7330518

[ref13] Guerra A. M. Lira A. Lameirao A. Selaru A. Abreu G. Lopes P.. (2020). Multiplicity of Carbapenemase-Producers Three Years after a KPC-3-Producing K. pneumoniae ST147-K64 Hospital Outbreak. Antibiotics 9:806. doi: 10.3390/antibiotics9110806, PMID: 33202755 PMC7696612

[ref14] Logan L. K. Weinstein R. A. (2017). The epidemiology of Carbapenem-resistant Enterobacteriaceae: The impact and evolution of a global menace. J. Infect. Dis. 215(suppl. 1), S28–S36. doi: 10.1093/infdis/jiw282, PMID: 28375512 PMC5853342

[ref15] Lopes E. Saavedra M. J. Costa E. de Lencastre H. Poirel L. Aires-de-Sousa M. (2020). Epidemiology of carbapenemase-producing Klebsiella pneumoniae in northern Portugal: predominance of KPC-2 and OXA-48. J. Glob. Antimicrob. Resist. 22, 349–353. doi: 10.1016/j.jgar.2020.04.007, PMID: 32348902

[ref16] Machado E. Canton R. Baquero F. Galan J. C. Rollan A. Peixe L.. (2005). Integron content of extended-spectrum-beta-lactamase-producing *Escherichia coli* strains over 12 years in a single hospital in Madrid, Spain. Antimicrob Agents Chemother 49, 1823–1829. doi: 10.1128/AAC.49.5.1823-1829.2005, PMID: 15855502 PMC1087637

[ref17] Magiorakos A. P. Burns K. Rodriguez Bano J. Borg M. Daikos G. Dumpis U.. (2017). Infection prevention and control measures and tools for the prevention of entry of carbapenem-resistant Enterobacteriaceae into healthcare settings: guidance from the European Centre for Disease Prevention and Control. Antimicrob. Resist. Infect. Control 6:113. doi: 10.1186/s13756-017-0259-z, PMID: 29163939 PMC5686856

[ref18] Magiorakos A. P. Srinivasan A. Carey R. B. Carmeli Y. Falagas M. E. Giske C. G.. (2012). Multidrug-resistant, extensively drug-resistant and pandrug-resistant bacteria: an international expert proposal for interim standard definitions for acquired resistance. Clin. Microbiol. Infect. 18, 268–281. doi: 10.1111/j.1469-0691.2011.03570.x, PMID: 21793988

[ref19] Manageiro V. Romao R. Moura I. B. Sampaio D. A. Vieira L. Ferreira E.. (2018). Molecular epidemiology and risk factors of Carbapenemase-producing Enterobacteriaceae isolates in Portuguese hospitals: results From European survey on Carbapenemase-producing Enterobacteriaceae (EuSCAPE). Front. Microbiol. 9:2834. doi: 10.3389/fmicb.2018.02834, PMID: 30538682 PMC6277554

[ref20] Masgala A. Kostaki K. Ioannnidis I. (2015). Multi drug resistant gram negative pathogens in long term care facilities: ASteadily arising problem. J. Infect. Dis. Diagno. 1:101. doi: 10.4172/2576-389X.1000124

[ref21] Mendes A. C. Novais A. Campos J. Rodrigues C. Santos C. Antunes P.. (2018). Mcr-1 in Carbapenemase-producing Klebsiella pneumoniae with hospitalized patients, Portugal, 2016-2017. Emerg. Infect. Dis. 24, 762–766. doi: 10.3201/eid2404.171787, PMID: 29553327 PMC5875258

[ref22] Millar M. (2020). “A capability perspective on antibiotic resistance, inequality, and child development,” in Ethics and Drug Resistance: Collective Responsibility for Global Public Health Public Health Ethics Analysis. eds. Jamrozik E. Selgelid M. (Cham, Switzerland: Springer).

[ref23] Miro E. Aguero J. Larrosa M. N. Fernandez A. Conejo M. C. Bou G.. (2013). Prevalence and molecular epidemiology of acquired AmpC beta-lactamases and carbapenemases in Enterobacteriaceae isolates from 35 hospitals in Spain. Eur. J. Clin. Microbiol. Infect. Dis. 32, 253–259. doi: 10.1007/s10096-012-1737-0, PMID: 22956023

[ref24] Mody L. Foxman B. Bradley S. McNamara S. Lansing B. Gibson K.. (2018). Longitudinal assessment of multidrug-resistant organisms in newly admitted nursing facility patients: implications for an evolving population. Clin. Infect. Dis. 67, 837–844. doi: 10.1093/cid/ciy194, PMID: 29635360 PMC6117444

[ref25] Navon-Venezia S. Kondratyeva K. Carattoli A. (2017). Klebsiella pneumoniae: a major worldwide source and shuttle for antibiotic resistance. FEMS Microbiol. Rev. 41, 252–275. doi: 10.1093/femsre/fux013, PMID: 28521338

[ref26] Nordmann P. (2014). Carbapenemase-producing Enterobacteriaceae: overview of a major public health challenge. Med. Mal. Infect. 44, 51–56. doi: 10.1016/j.medmal.2013.11.007, PMID: 24360201

[ref27] Nordmann P. Poirel L. (2013). Strategies for identification of carbapenemase-producing Enterobacteriaceae. J. Antimicrob. Chemother. 68, 487–489. doi: 10.1093/jac/dks426, PMID: 23104494

[ref28] Ortega A. Saez D. Bautista V. Fernandez-Romero S. Lara N. Aracil B.. (2016). Carbapenemase-producing *Escherichia coli* is becoming more prevalent in Spain mainly because of the polyclonal dissemination of OXA-48. J. Antimicrob. Chemother. 71, 2131–2138. doi: 10.1093/jac/dkw148 27147304

[ref29] Pellegrini C. Mercuri P. S. Celenza G. Galleni M. Segatore B. Sacchetti E.. (2009). Identification of Bla(IMP-22) in pseudomonas spp. in urban wastewater and nosocomial environments: biochemical characterization of a new IMP metallo-enzyme variant and its genetic location. J. Antimicrob. Chemother. 63:901. doi: 10.1093/jac/dkp061, PMID: 19270313

[ref30] Pena I. Picazo J. J. Rodriguez-Avial C. Rodriguez-Avial I. (2014). Carbapenemase-producing Enterobacteriaceae in a tertiary hospital in Madrid, Spain: high percentage of colistin resistance among VIM-1-producing Klebsiella pneumoniae ST11 isolates. Int. J. Antimicrob. Agents 43, 460–464. doi: 10.1016/j.ijantimicag.2014.01.021, PMID: 24657043

[ref31] Perdigao J. Modesto A. Pereira A. L. Neto O. Matos V. Godinho A.. (2019). Whole-genome sequencing resolves a polyclonal outbreak by extended-spectrum beta-lactam and carbapenem-resistant Klebsiella pneumoniae in a Portuguese tertiary-care hospital. Microb. Genom 7:349. doi: 10.1099/mgen.0.000349, PMID: 32234124 PMC8627661

[ref32] Pires J. Novais A. Peixe L. (2013). Blue-carba, an easy biochemical test for detection of diverse carbapenemase producers directly from bacterial cultures. J. Clin. Microbiol. 51, 4281–4283. doi: 10.1128/JCM.01634-13, PMID: 24108615 PMC3838089

[ref33] Pitout J. D. Nordmann P. Poirel L. (2015). Carbapenemase-producing Klebsiella pneumoniae, a key pathogen set for global nosocomial dominance. Antimicrob. Agents Chemother. 59, 5873–5884. doi: 10.1128/AAC.01019-15, PMID: 26169401 PMC4576115

[ref34] Poirel L. Walsh T. R. Cuvillier V. Nordmann P. (2011). Multiplex PCR for detection of acquired carbapenemase genes. Diagn. Microbiol. Infect. Dis. 70, 119–123. doi: 10.1016/j.diagmicrobio.2010.12.002, PMID: 21398074

[ref35] Queenan A. M. Bush K. (2007). Carbapenemases: the versatile beta-lactamases. Clin Microbiol Rev 20, 440–458. doi: 10.1128/CMR.00001-07 17630334 PMC1932750

[ref36] Rodrigues C. Novais A. Machado E. Peixe L. (2014). Detection of VIM-34, a novel VIM-1 variant identified in the intercontinental ST15 Klebsiella pneumoniae clone. J. Antimicrob. Chemother. 69, 274–275. doi: 10.1093/jac/dkt314, PMID: 23934739 PMC7314032

[ref37] Teixeira J. V. Cecilio P. Goncalves D. Vilar V. J. Pinto E. Ferreira H. N. (2016). Multidrug-resistant Enterobacteriaceae from indoor air of an urban wastewater treatment plant. Environ. Monit. Assess. 188:388. doi: 10.1007/s10661-016-5382-4, PMID: 27260528

[ref38] Tenover F. C. Arbeit R. D. Goering R. V. Mickelsen P. A. Murray B. E. Persing D. H.. (1995). Interpreting chromosomal DNA restriction patterns produced by pulsed-field gel electrophoresis: criteria for bacterial strain typing. J. Clin. Microbiol. 33, 2233–2239. doi: 10.1128/jcm.33.9.2233-2239.1995, PMID: 7494007 PMC228385

[ref39] Viedma E. Juan C. Villa J. Barrado L. Orellana M. A. Sanz F.. (2012). VIM-2-producing multidrug-resistant *Pseudomonas aeruginosa* ST175 clone, Spain. Emerg. Infect. Dis 18, 1235–1241. doi: 10.3201/eid1808.111234, PMID: 22840969 PMC3414013

[ref40] Vubil D. Figueiredo R. Reis T. Canha C. Boaventura L. Das G. J. (2017). Outbreak of KPC-3-producing ST15 and ST348 Klebsiella pneumoniae in a Portuguese hospital. Epidemiol. Infect. 145, 595–599. doi: 10.1017/S0950268816002442, PMID: 27788691 PMC9507641

[ref41] WHO (2015). Global Action Plan on Antimicrobial Resistance. Available at: http://www.who.int/antimicrobial-resistance/publications/global-action-plan/en/ (Accessed April 27, 2021).

