# Supplementary material for: Intra- and Extra-Hospital Dissemination of IMP-22-Producing Klebsiella pneumoniae in Northern Portugal: The Breach of the Hospital Frontier Toward the Community
Source: Front Microbiol. 2021 Dec 14;12:777054. doi: 10.3389/fmicb.2021.777054 (PMC8713047; doi:10.3389/fmicb.2021.777054)
Supplement: Supplementary file 1 [file Table_1.docx]

Supplementary Material

Intra and Extra-hospital dissemination of IMP-22 producing *Klebsiella pneumonia*e in Northern Portugal: the breach of the hospital frontier towards the community

Daniela Gonçalves^1-4 #^, Pedro Cecílio^1 #^, Alberta Faustino^5^, Carmen Iglesias^5^, Fernando Branca^5^, Alexandra Estrada^5^ and Helena Ferreira^1,2 *^

^1^Microbiology Laboratory - Biological Sciences Department, Faculty of Pharmacy, University of Porto, Porto, Portugal;

^2^UCIBIO - Research Unit on Applied Molecular Biosciences, REQUIMTE, Porto, Portugal;

^3^ISAVE - Instituto Superior de Saúde, Amares, Portugal;

^4^CICS - Interdisciplinary Centre in Health Sciences, Amares, Portugal;

^5^Clinical Pathology Service - Braga Hospital, Braga, Portugal;

^#^These authors contributed equally to this study.

*** Correspondence:**Helena Maria Neto Ferreira de Sousa

[hferr@ff.up.pt](mailto:hferr@ff.up.pt)

**Table S1. MICs used to interpret β-lactam and non-β-lactam antibiotic susceptibility in the context of *K. pneumoniae* clinical isolates**

| **Group** | **Antimicrobial agent** | **MICs interpretative criteria (µg/ml)** | | |
| --- | --- | --- | --- | --- |
|  |  | **S** | **I** | **R** |
| Penicillins | Ampicillin | ≤8 | 16 | ≥32 |
|  | Piperacillin | ≤16 | 32-64 | ≥128 |
|  | Ticarcillin | ≤16 | 32-64 | ≥128 |
| β-lactam/β-lactamase inhibitor combination | Amoxicillin + clavulanic acid | ≤8/4 | 16/8 | ≥32/16 |
|  | Piperacillin + tazobactam | ≤16/4 | 32/4-64/4 | ≥128/4 |
|  | Ticarcillin + clavulanic acid | ≤16/2 | 32/2-64/2 | ≥128/2 |
| Cephems | Cephalothin | ≤8 | 16 | ≥32 |
|  | Cefuroxime | ≤8 | 16 | ≥32 |
|  | Ceftazidime | ≤4 | 8 | ≥16 |
|  | Cefotaxime | ≤1 | 2 | ≥4 |
|  | Cefepime | ≤8 | 16 | ≥32 |
| Monobactams | Aztreonam | ≤4 | 8 | ≥16 |
| Carbapenems | Imipenem | ≤1 | 2 | ≥4 |
|  | Ertapenem | ≤0,5 | 1 | ≥2 |
|  | Meropenem | ≤1 | 2 | ≥4 |
| Aminoglycosides | Gentamicin | ≤4 | 8 | ≥16 |
|  | Tobramycin | ≤4 | 8 | ≥16 |
|  | Amikacin | ≤16 | 32 | ≥64 |
| Tetracyclines | Minocycline | ≤4 | 8 | ≥16 |
| Fluoroquinolones | Ciprofloxacin | ≤1 | 2 | ≥4 |
|  | Levofloxacin | ≤2 | 4 | ≥8 |
|  | Pefloxacin | ≤1 | 2 | ≥4 |
| Folate pathway inhibitors | Trimethoprim + sulfamethoxazole | ≤2/38 | - | ≥4/76 |
| Nitrofurans | Nitrofurantoin | ≤32 | 64 | ≥128 |
| Rifamycins | Rifampicin | ≤1 | 2 | ≥4 |

**Note:** According to the CLSI criteria.

**Table S2. Criteria used to interpret β-lactam and non-β-lactam antibiotics susceptibility in the context of *K. pneumoniae* intestinal isolates**

| **Group** | **Antimicrobial agent** | **Zone diameter interpretative criteria**  **(mm)** | | |
| --- | --- | --- | --- | --- |
|  |  | **S** | **I** | **R** |
| Penicillins | Ampicillin | ≥17 | 14-16 | ≤13 |
| β-lactam/β-lactamase inhibitor combination | Amoxicillin + clavulanic acid | ≥18 | 14-17 | ≤13 |
| Cephems | Ceftazidime | ≥21 | 18-20 | ≤17 |
|  | Cefotaxime | ≥26 | 23-25 | ≤22 |
|  | Cefepime | ≥18 | 15-17 | ≤14 |
| Cephamycins | Cefoxitin | ≥18 | 15-17 | ≤14 |
| Monobactams | Aztreonam | ≥21 | 18-20 | ≤17 |
| Carbapenems | Imipenem | ≥23 | 20-22 | ≤19 |
|  | Ertapenem | ≥22 | 19-21 | ≤18 |
|  | Meropenem | ≥23 | 20-22 | ≤19 |
| Aminoglycosides | Gentamicin | ≥15 | 13-14 | ≤12 |
|  | Tobramycin | ≥15 | 13-14 | ≤12 |
|  | Amikacin | ≥17 | 15-16 | ≤14 |
|  | Netilmicin | ≥15 | 13-14 | ≤12 |
|  | Streptomycin | ≥15 | 12-14 | ≤11 |
| Tetracyclines | Tetracycline | ≥15 | 12-14 | ≤11 |
| Fluoroquinolones | Nalidixic acid | ≥19 | 14-18 | ≤13 |
|  | Ciprofloxacin | ≥21 | 16-20 | ≤15 |
| Folate pathway inhibitors | Trimethoprim + sulfamethoxazole | ≥16 | 11-15 | ≤10 |
| Phenicols | Chloramphenicol | ≥18 | 13-17 | ≤12 |
| Nitrofurans | Nitrofurantoin | ≥17 | 15-16 | ≤14 |
| Glycylcyclines | Tigecycline* | ≥18 | - | ≤15 |

**Note:** According to the CLSI or EUCAST (*) criteria (<http://www.eucast.org/clinicalbreakpoints/>).
